# A Multilevel Meta-analysis of Single-Case Research on Interventions for Internalizing Disorders in Children and Adolescents

**DOI:** 10.1007/s10567-023-00432-9

**Published:** 2023-04-03

**Authors:** Marija Maric, Lea Schumacher, Wim Van den Noortgate, Linda Bettelli, Wies Engelbertink, Yvonne Stikkelbroek

**Affiliations:** 1 Developmental Psychology, University of Amsterdam, Amsterdam, The Netherlands; 2 Research Institute of Child Development and Education, University of Amsterdam, Amsterdam, The Netherlands; 3 Medical Psychology, University Medical Center Hamburg-Eppendorf, Hamburg, Germany; 4 Faculty of Psychology and Educational Sciences & Itec, an Imec Research Group, KU Leuven, Leuven, Belgium; 5 Child and Adolescent Studies, Utrecht University, Utrecht, The Netherlands; 6 Depression Expert Center for Youth, Mental Health Care Oost-Brabant, Boekel, The Netherlands

**Keywords:** Internalizing disorders, Children and adolescents, Meta-analysis, Single case

## Abstract

The effectiveness of interventions for internalizing disorders in children and adolescents was studied using a review and meta-analysis of published single-case research. Databases and other resources were searched for quantitative single-case studies in youth with anxiety, depressive, and posttraumatic stress disorders. Raw data from individual cases were aggregated and analyzed by means of multilevel meta-analytic models. Outcome variables were symptom severity assessed across baseline and treatment phases of the studies, and diagnostic status at post- and follow-up treatment. Single-case studies were rated for quality. We identified 71 studies including 321 cases (*M*^*age*^ = 10.66 years; 55% female). The mean quality of the studies was rated as below average, although there were considerable differences between the studies**.** Overall, positive within-person changes during the treatment phase in comparison to the baseline phase were found. In addition, positive changes in the diagnostic status were observed at post- and follow-up treatment. Yet high variability in treatment effects was found between cases and studies. This meta-analysis harvests the knowledge from published single-case research in youth-internalizing disorders and illustrates how within-person information from single-case studies can be summarized to explore the generalizability of the results from this type of research. The results emphasize the importance of keeping account of individual variability in providing and investigating youth interventions.

**Supplementary Information:**

The online version contains supplementary material available at 10.1007/s10567-023-00432-9.

## Introduction

Internalizing disorders such as anxiety, depressive, and posttraumatic stress disorders (ADs, DD and PTSD), are among the most common mental health problems in children and adolescents (Merikangas et al., 2010), and their co-occurrence is high (McElroy & Patalay, 2019). Numerous empirically established interventions are available for treating these disorders (e.g., Crowe & McKay, 2017; Oud et al., 2019; Weems & Neill, 2020; Weisz et al., 2017). Evidence base underlying these treatments was built upon Randomized Controlled Trials (RCTs) in which average symptom scores of one group of participants are compared to those of a group of participants in a different condition. However, treatment effects that evolve within persons might not always be captured by between-person comparisons (e.g., Maric et al., 2012; Schuurman, 2023). There is nowadays a shared understanding that, next to RCTs, we need more idiographic types of research methods that can capture changes in individual risk factors, symptoms, and treatment goals while at the same time maintaining methodological rigor.

Quantitative single-case research is increasingly recognized as a valuable way to test within-person treatment effects in youth populations, both as an add-on for RCTs and as a stand-alone method (Kazdin, 2019; Maric, et al., 2012). International guidelines consider evidence gained from this type of research as one of the most rigorous forms of evidentiary support for therapies (Onghena et al., 2019). Further, single-case research can be a first step in testing innovative interventions prior to investigations in costly and time-intensive RCTs (Gaynor & Harris, 2008; Maric et al., 2012). In youth with internalizing disorders and specific comorbidities, single-case research can be implemented as a stand-alone method when collecting large data would be unfeasible within the time limits of the research project. Finally, using single-case methods existing treatment protocols for youth-internalizing disorders can be implemented in real-world clinical practice and the formats and conditions under which they are effective can be tested.

While it is tremendously important to study within-person effects to evaluate treatments, the reach and impact of these studies remain limited. This is partly due to the observation that single-case studies in youth interventions for anxiety, depression, and trauma often include a small number of cases. Even if the research questions are considered valuable for the field and an appropriate single-case design is used, questions about the strength of this evidence and the scope of implications of the results remain. Harvesting knowledge from these individual single-case studies is a next important step in order to broaden our knowledge about effects of youth interventions. While this rising number of single-case studies provides valuable information on within-person treatment effects, it is unlikely that they will strongly impact the knowledge on youth treatments unless their results are integrated. A meta-analysis of single-case research data permits researchers to synthesize the results of published studies quantitively to further help determining an evidence-base for therapies for internalizing disorders in children and adolescents (Dowdy et al., 2021; Onghena, Michiels, Jamshidi, Moeyaert, & Van den Noortgate, 2018; Van den Noortgate & Onghena, 2003). While in RCTs, between-person effects are examined, and meta-analyses of RCTs concern information on the sample level, meta-analyses of single-case research involve during-treatment within-person changes and data, and treatment effects on the case level are investigated. Few single-case research meta-analyses exist so far. Exceptions are for instance Richman et al. (2015), who investigated effects of treatment on non-social behavior in individuals, and Heyvaert et al. (2012), who evaluated intervention effectiveness for reducing challenging behavior in individuals with intellectual disabilities. To our knowledge, no meta-analysis of published single-case research exist in the area of youth psychopathology.

A strength of single-case research is that it can assess the effect for specific cases, for specific treatments, in specific contexts. Despite this ideosyncraticity, we expect that there is some communality, e.g., if a certain effect was found effective for some cases, it may be expected effective for other cases as well. A meta-analysis allows us to investigate whether there is an overall effect of the intervention, across all cases and studies. Second, it allows to quantify to what extent the effect varies between studies and cases, i.e., to what extent the effects found can be generalized and how much heterogeneity in treatment effects is present. Third, if there is heterogeneity in treatment effects, it allows to explore whether we can explain this variation by exploring the moderating effects of case and study characteristics. In single-case meta-analysis, treatment effects are estimated for each individual, offering opportunities to study moderating effects of case characteristics, in contrast to group comparisons in (meta-analyses of) RCTs. As, to our knowledge, no meta-analysis on this topic has been conducted, the overall within-person treatments effects, its heterogeneity, and moderators that could potentially explain the heterogeneity are unknown for treatments of internalizing disorders in youth.

This meta-analysis was driven by the fact that evidence of treatments for youth-internalizing disorders is mainly based on information on between-person effects from RCTs and the urge for information on within-person treatment effects and methods to study these. Further, while single-case studies are an acknowledged phenomenon in youth-internalizing intervention research, it is high time to start harvesting results of quantitative single-case research by systematically and quantitavely integrating its findings. Recent methodological developments, and collaborations between clinical researchers and methodologists make this endeavor possible. The aim of this meta-analysis is to provide a broad overview of the field and assess the overall within-person treatment effects, its heterogeneity and moderators that could explain this heterogeneity. As the single-case literature on internalizing disorders has not even been systematically reviewed yet, this study is exploratory in nature.

## Methods

The planned analyses were pre-registered on OSF: https://osf.io/zjswg/?view_only=2b14c0d282e94a849f9d741a5cf759a1. Due to the unkown number and nature of potentially included studies, the analysis plan was pre-registered after the search and during data extraction. The meta-analysis was conducted according to the PRISMA guidelines (Page et al., 2021).

### Literature Search

We searched for quantitative single-case studies in three databases: PsycInfo, Medline, and Web of Science, without a lower limit for the date. The title, abstract, keywords, and subject headings were searched on January 6th 2020 with search terms from three categories: #1 Single-Case Experimental Studies, #2 Children and Adolescents, and #3 Internalizing and Externalizing Problems. During the screening process, the inclusion criteria were refined to only include internalizing disorders (not externalizing disorders), as a sufficient number of studies could be found on internalizing disorders in order to do a meta-analysis. This focus also made that we could limit the heterogenity between the studies to some extent. To include all current studies, a search was done in PsycInfo, Medline, and Web of Science with refined search criteria only including internalizing disorders in May 2021 for the period January 2020 to May 2021 and in February 2023 for the period May 2021 to February 2023. A full list of the original and refined search terms can be found in Supp1. In addition, Google scholar was checked for articles that were potentially missed and the references of all included studies were screened for possibly relevant studies. PsycArxiv and OSF were searched for gray literature in January 2021 and in February 2023.

### Study Selection

For the current meta-analysis, inclusion criteria were as follows: a quantitative Single-Case (Experimental) Design [SC(E)D] was used; participants were children (4 through 17 years old) who at the start of the study met DSM criteria for anxiety, depression or posttraumatic stress disorder; who received treatment aimed at reduction of internalizing symptoms; and results on symptom severity or diagnostic status were reported at least at one assessment point before and one assessment point after the treatment. We included studies involving both experimental, quasi-experimental, and non-experimental single-case designs as we wanted to safeguard power of these analyses and provide a complete overview of SC(E)D research on internalizing disorders in youth. Cases with an intellectual disability or an IQ below 70 and cases with a medical condition were excluded.

The abstracts and the full texts of the studies were screened with the inclusion criteria by two independent raters. 20% of all abstracts and all full texts were double screened. Disagreement was resolved during discussion, through thorough checking of the inclusion criteria.

### Outcome Variables and Moderators

The repeatedly assessed symptom severity across phases as depicted in individual graph data was the main outcome variable in this study. In most studies, only one main outcome variable was present. In studies where several variables were presented as outcome variables in graph data, one outcome variable was selected for the purpose of this meta-analysis, using the following criteria: (a) variable was related to the primary diagnosis (e.g., anxiety symptoms were chosen above comorbid ADHD symptoms); and (b) the same variables were assessed across different studies (e.g., spontaneous speech was assessed in selective mutism studies). In most studies, main outcome variable was self-reported by the child [45% in anxiety disorders (AD), 65% in major depressive disorder (MDD), 75% in posttraumatic stress disorder (PTSD) studies]. Parent-reported child symptoms were present in 42% of AD studies, 15% of MDD studies, and 12% of PTSD studies. The remainder of the outcome variables was rated by teachers or independent raters. Overall, behavioral outcome variables were parent and other reported (e.g., speech in class, separation from parents). An overview of variables indicating symptom severity included in the analyses can be found in Supp3.

Primary diagnosis (AD, MDD, or PTSD), according to DSM III, IV, or 5, of the cases, on pre-, post-, and follow-up treatment was included as a categorical outcome variable (yes/no).

Five potential moderators identified as important in previous studies on youth interventions (Maric et al., 2015) were tested: age, disorder category (AD, MDD, PTSD), target group (children, parents, parents and children), sample type (referred, recruited, referred and recruited, other), and treatment dosage (operationalized as number of sessions).

### Data Extraction

Extraction of study characteristics, demographic, diagnosis, and treatment information was done independently by two raters. Graph data were extracted with DigitizeIt software 2.5 (*DigitizeIt*, 2021; Rakap et al., 2016). Extraction of graph data was done by two independent raters, and 30% percent of the studies were cross checked. Finally, post- and follow-up diagnosis data were extracted. Prior to analyses, extracted data, and case characteristics were cross checked.

### Quality Rating of the Studies

Studies were rated into non-experimental, quasi-experimental, and experimental single-case designs by MM (Tate et al., 2016; Supp3). Quality was assessed using 15-item Risk of Bias in N-of-1 Trials (RoBiNT) scale (Tate et al., 2013). It contains internal validity (IV; 7 items; e.g., ‘design’) and external validity and interpretation (EVI; 8 items; e.g., ‘therapeutic setting’). Items are rated on a 3-point scale (score range 0–2) with a maximum total score 30, and for the subscales 14 (IV) and 16 (EVI). Interrater reliability of RoBiNt scale between experienced raters ranges from 0.87 to 0.90, and from 0.93 to 0.95 between experienced and novice raters (Tate et al., 2013). YS and LB rated independently 30% of the studies resulting in interrater reliability (ICC) of 0.78, 0.78, and 0.72 for the total score, and IV and EVI subscale scores, respectively. Differences were resolved through discussions. Both YS and LB each rated half of the remaining 70% of the studies.

### Statistical Analyses

Instead of calculating summary effect sizes for each study, and combining these as in regular meta-analyses, we combined the raw data from all cases (Van den Noortgate & Onghena, 2008). For both the repeated assessments of symptom severity and post-/follow-up treatment diagnosis, the data were analyzed using multilevel regression models. All analyses were done in R 4.1.2 using the package *lme4* (Bates et al., 2015). Data and code can be found in Supp4/Supp5.

*Analysis of symptom severity* Several variables had to be (re)coded for the analysis of the repeated assessments of symptom severity. First, the phase variable was coded into baseline and treatment phase. For studies which included a baseline and two different treatment phases referring to the same therapies with, e.g., different intensity (e.g., Carlson, 1999) or different techniques from CBT (e.g., Nakamura, 2008), the two treatment phases were taken together. Due to a lack of available data across studies, it was decided to not include data from follow-up phases in this analysis.

Second, the time variable was coded such that it started at 0 at the beginning of the treatment (negative values were assigned to baseline phase) and increased with 1 for each additional week. Third, the scores indicating the symptom severity were reverse coded for some studies so that a score decrease implies improvement for each study. Finally, to be able to combine and compare scores between studies and cases, symptom severity scores were standardized as proposed by Van den Noortgate and Onghena (2008). This was done by estimating a two-level model for each study with phase (baseline phase, treatment phase), time, and their interactions as predictors, symptom severity as the outcome, and random effects for all predictors across cases. Subsequently, the original scores from each study were divided by the residual standard deviation of the model for this study. For studies with only one case, a linear regression model was estimated, and scores were divided by the estimated residual standard deviation. Finally, age was centered to ease interpretation for the moderator analysis.

To meta-analyze the (standardized) data from all cases, a three-level linear regression model with symptom severity as the dependent variable and phase (baseline phase, treatment phase), time, and their interaction as predictors was estimated. Here, the intercept and the effect of the time can be interpreted as the expected level at the end of the baseline phase, and the time trend in the baseline phase, whereas the effects of the phase and the interaction term can be interpreted as the immediate treatment effect at the start of the intervention, and the effect the treatment has on time trend (Van den Noortgate & Onghena, 2008). All four coefficients were allowed to vary randomly between cases and between studies. Furthermore, one model for each moderator (age, disorder type, target group, sample type, and treatment dosage) was estimated in which the moderator variable as well as its interaction with phase, with time, and with the interaction between phase and time were included as additional predictors.

*Analysis of diagnostic status* Due to inclusion criteria, all participants had a formal DSM diagnosis at pre-treatment. To assess the probability of a diagnosis at post-treatment and at follow-up, we conducted two-level logistic regressions with the diagnostic status (yes = 1, no = 0) at the respective time point as the outcome variable. First, an intercept model was estimated to evaluate the overall probability of a diagnosis, separately for post-treatment and for follow-up. Subsequently, for moderator analysis, three models were estimated with age, disorder category, and target group as respective predictors, again in separate analyses for diagnostic status at post-treatment and at follow-up. Due to limited amount of data for diagnosis data, we limited this analysis to the three most important moderators. For all models, the intercept was allowed to vary between studies.

## Results

**Fig. 1:**
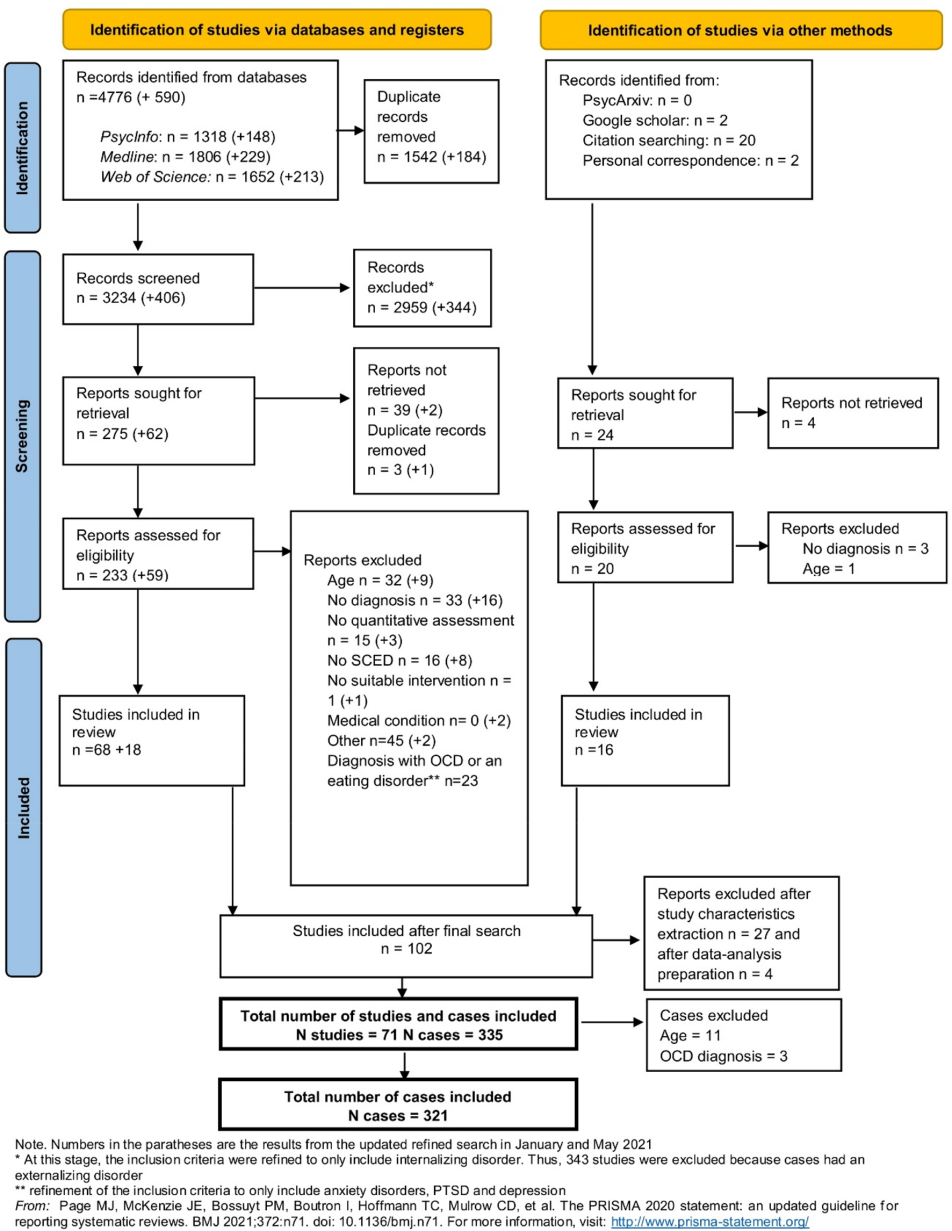
PRISMA flow diagram of included studies and cases

102 studies matched the inclusion criteria. 27 studies were excluded due to unclarity about diagnostic procedures or absence of information about treatment outcomes on the case level. Four additional studies were excluded as the data in these studies could not be standardized for the purposes of multilevel meta-analysis. Thus, a total of 71 studies were included. From these 71 included studies, we still had to exclude 14 individual cases as they did not fit inclusion criteria, leaving a total number of cases of 321. A detailed overview of the study inclusion process is presented in PRISMA flow chart (Fig. 1). An overview of the excluded studies can be found in Supp2.

### Study and Case Characteristics

A summary of study characteristics is presented in Supp3 wherein case-level information on all variables can be found. Number of cases per study ranged from 1 to 17 (*M* = 4.45). Total number of dropouts was 12 and ranged from 0 (48 studies) to 6 (1 study); 7 studies did not report on drop out. Mean age of the cases was 10.66 years (*SD* = 3.49; range 4–17); 55% of the cases were female (for about 50 cases gender was not reported or only on a sample level). For about 30% of the cases, no information regarding ethnicity or cultural background of participants was reported (Supp3). In 22% of the cases, no information about comorbidity was reported. In 18% of the cases, no comorbidity was observed. Comorbidity with another internalizing disorder was present in almost 40% of the cases, with an externalizing disorder in 4%, and with ADHD and ASS in 18% and 4% of the cases, respectively. 4% of the cases had other comorbid disorder (e.g., learning, speech, sleeping disorder).

Length of the treatment in weeks ranged between 0.02 (3-h treatment) and 68 weeks. On average, the treatments consisted of 10.47 sessions, ranging from 1 to 36 sessions. Overall, average scores for the total RoBiNT scale, and internal and external validity subscales were 12.06 (*SD* = 3.85; score range 4–21), 3.17 (*SD* = 2.52; score range 0–9), and 8.89 (*SD* = 2.26; score range 4–14), respectively. In Supp3, quality scores of (sub)scale(s) for each study can be found.

### Within-Person Symptom Change

*Average treatment effect*: 4,153 datapoints from 222 cases from 47 studies were available. The first 3-level regression analysis with phase, time, and their interaction as predictors indicated that, on average, there was (across cases and studies) a significant immediate reduction of the symptom severity at the start of the treatment phase; *b* = − 0.67, 95% CI = [− 1.10; − 0.24], *p* = 0.002. The symptom severity significantly reduced with 0.14 standard deviation per week during the baseline phase; *b* = − 0.14, 95% CI = [− 0.24; − 0.03], *p* = 0.031. The linear decrease in symptoms became more pronounced during the treatment phase when compared to the baseline phase as indicated by the interaction between phase and time; *b* = − 0.27, 95% CI = [− 0.47–0.07], *p* = 0.005, resulting in a reduction of 0.41 (= − 0.14–0.27) standard deviation per week during the treatment phase. These analyses were re-done excluding three studies that were concerned with medication treatment and one study concerned with animal-assisted therapy, respectively (Table 1). In both cases, the conclusions remained the same. Similarly, we repeated this analysis when only including studies with experimental designs. Again the conclusions remained the same.^1^

**Table 1:** Summary of study and case characteristics (*N* = 71 studies; 321 cases)

	N of studies
World part	
North America	48
Europe	13
Australia and New Zealand	8
South America	1
Asia	1
Type of diagnosis^a^	
AD	52 [*n* cases = 231]
MDD	8 [*n* cases = 35]
PTSD	11 [*n* cases = 55]
Type of sample	
Referred	41
Recruited	22
Combined referred and recruited	1
Other^b^	2
NR	5
Setting	
University clinic (UC)	34
Outpatient psychiatric clinic (OPC)	18
OPC/UC	1
School	5
Other^c^	4
NR	9
Type of treatment	
CBT-oriented	59
CBM	3
Medication	3
Parent–child interaction therapy	2
Other^d^	4
Treatment target group^e^	
Child and parent	33
Child-only	33
Parent-only	5
Type of SC(E)D	
Experimental	
Multiple baseline	41
Changing-criterion	2
Alternating-treatments	1
Quasi-experimental	
AB	8
Non-experimental	
Pre-post	11
One-phase design	8

*AD* Anxiety Disorders; *MDD* Major Depressive Disorder; *PTSD* Post Traumatic Stress Disorder; *CBT* Cognitive Behavioral Treatment; *CBM* Cognitive Bias Modification. *NR* not reported

^a^Determined via semi-structured diagnostic interviews (87% of the cases), via clinical interview (screening; 12% of the cases). In four cases both diagnostic interview and screening were used. In one case information about the method was unavailable, but it was clearly stated that the participant had a formal DSM diagnosis and was hospitalized for that

^b^Moved from another trial

^c^Schools, community services, hospital

^d^Acceptance and Commitment Therapy, Interpersonal Psychotherapy, Mindfulness, Equine-assisted trauma therapy

^e^Four studies had mixed target groups for their cases

**Fig. 2:**
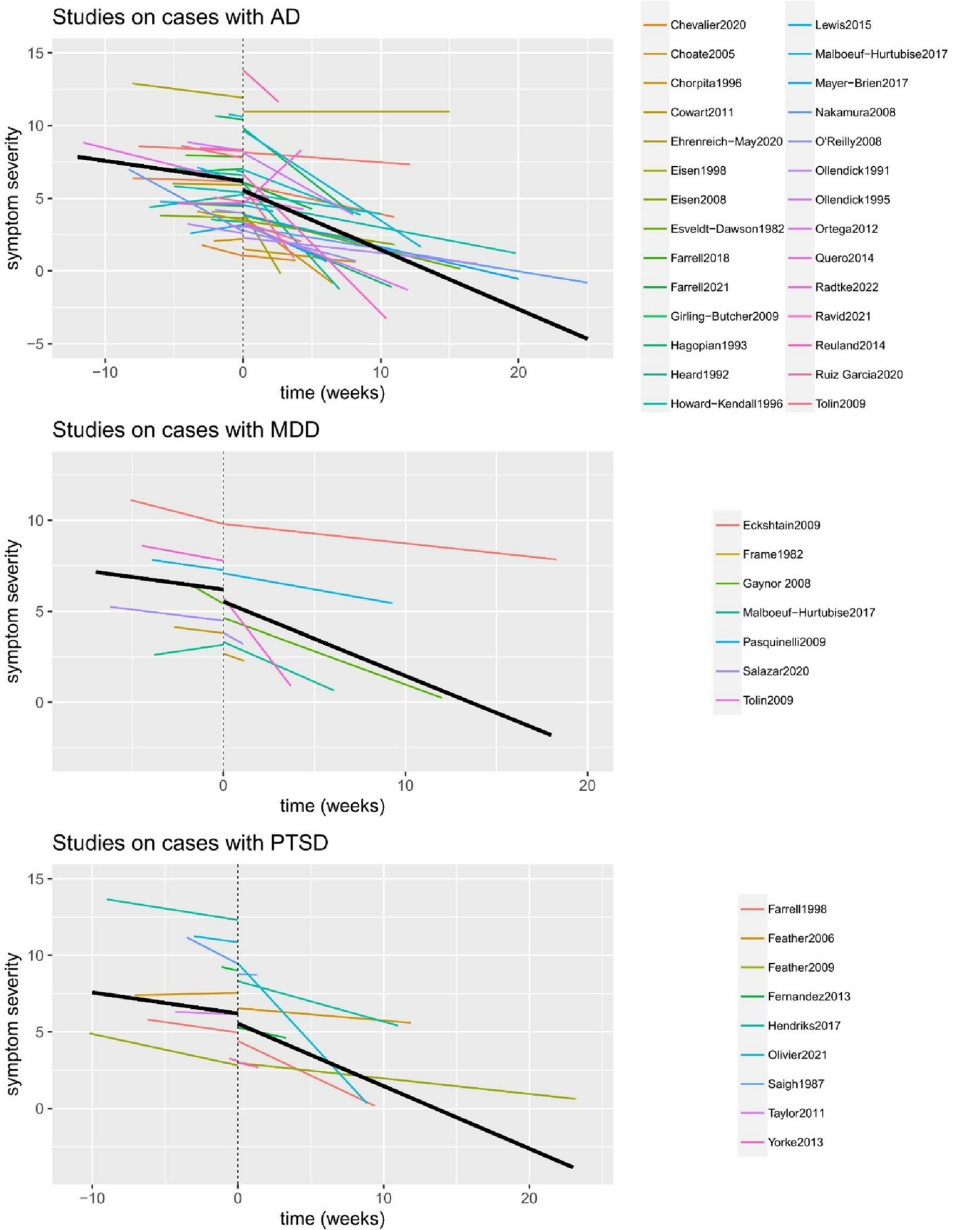
Estimated effects for the symptom development during baseline and treatment phase for each study *Note* The black line represents the average effects across all studies; for time < 0 the lines represent the estimated slope during the baseline phase, for time > 0 the lines represent the estimated slope during the treatment phase; the “drop” at *t* = 0 indicates the immediate symptom reduction at treatment start; symptom severity was standardized across studies and values < 0 mean no symptoms anymore; the time interval varied between studies. Estimates are empirical Bayes estimates

**Fig. 3:**
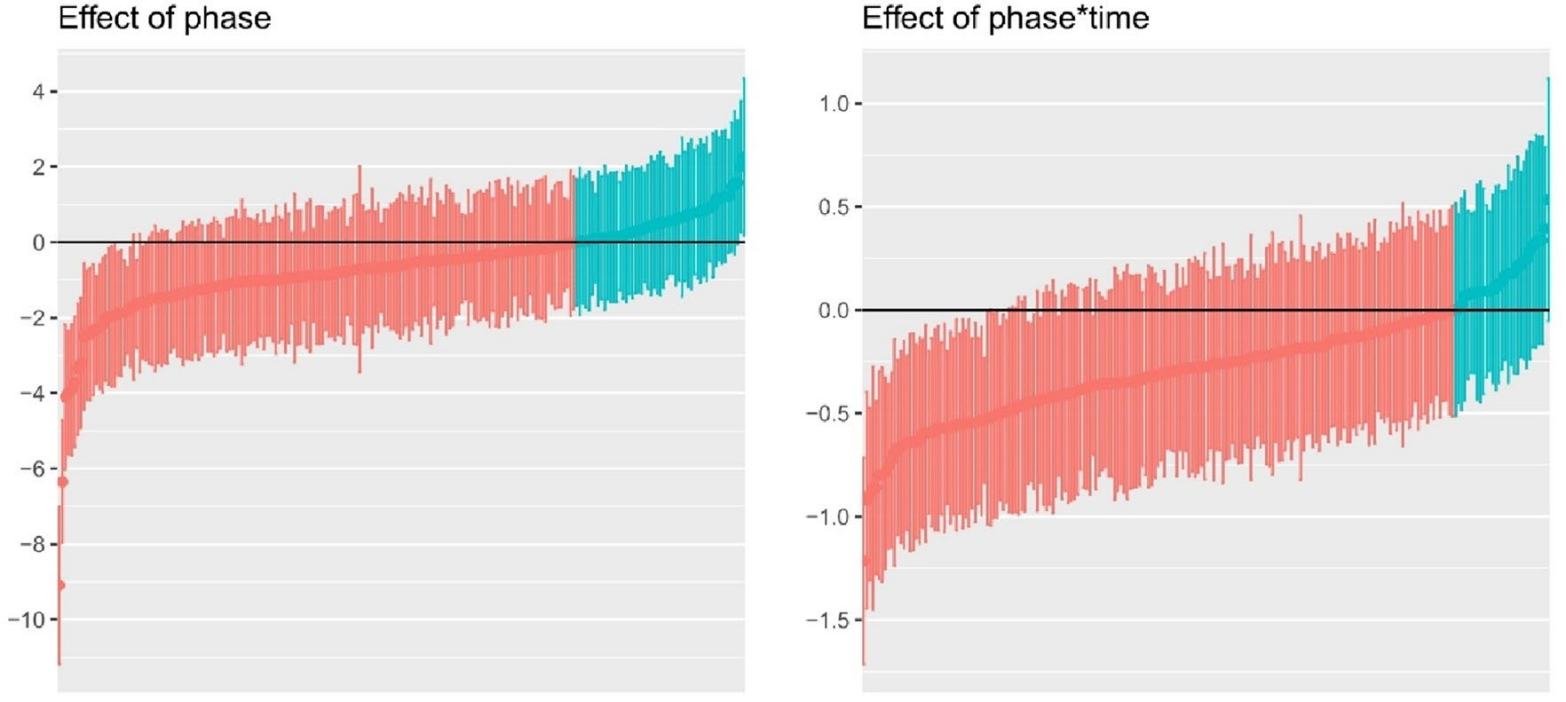
Estimated treatment effect for each individual case *Note* Each dot represents the empirical Bayes estimate of the effect for each individual case, lines represent the corresponding 95% confidence intervals

*Heterogeneity of the treatment effect* The symptom development for each study during the baseline and the treatment phase is depicted in Fig. 2, and the estimated effects for all individual cases are depicted in Fig. 3. Compared to the variation within subjects (*σ* = 0.94), there was much variation for the intercept between the studies (the estimated standard deviation *τ*  = 3.17) and cases (*τ* = 2.19). This shows that the symptom severity was very different between cases and studies at treatment start. Also the treatment effects varied considerably between studies and cases. Between the studies, the effect of phase (i.e., the immediate reduction in symptom severity at treatment start) varied with *τ* = 1.14 and the effect of phase*time (i.e., difference in symptom reduction between the baseline and treatment phase) varied with *τ* = 0.59. Given the assumption that effects are normally distributed across studies, this would mean that for 95% of the studies, the immediate treatment effect ranges between − 2.90 and 1.56, and that the effect is negative for 72% of the studies. For the effect on the time trend (phase*time), the 95% prediction interval is [− 1.43; 0.89] with a negative effect for 68% of the studies. Between cases, the effect of phase varied with an estimated standard deviation *τ* = 1.49 and the effect of phase*time with *τ* = 0.36. These results suggest that for 95% of the cases and a typical study, the immediate effect varies between − 3.59 and 2.25 (with 67% of the effects being negative), and the effect of phase*time varies between -0.98 and 0.44, and that the effect is negative for about 77% of the cases. Figure 3 visualizes that despite large variability in the individual treatment effects, for the majority of cases a reduction is expected in symptom severity as a response to the treatment, although for a minority of cases, this reduction is also statistically significant. Across cases, there is a large negative correlation between the random intercept and the random effects of the interaction between phase and time, *r* = − 0.75. This indicates that the larger the symptom severity at the end of the baseline, the more pronounced is the symptom severity reduction in the treatment phase compared to the baseline phase.

*Moderators* The results for the three-level regressions which include the moderator variables are displayed in Supp6. Almost none of the variables showed a significant effect on the baseline level or trend in symptom severity, nor on the immediate effect of time trend (interaction with phase and time respectively). Only the immediate symptom reduction during the treatment phase seems to be more pronounced for cases with PTSD when compared to cases with AD or MDD; *b* = − 1.16 [− 2.19; − 0.14], *p* = 0.027.

### Diagnostic Status at Post-Treatment and Follow-up

Data on diagnostic status were available for 268 cases from 62 studies at post-treatment and 191 cases from 44 studies at follow-up. At post-treatment, 28.46% (*n* = 76) cases still met diagnostic criteria for AD, MDD, or PTSD. At follow-up, 21.99% (*n* = 42) cases still met diagnostic criteria for AD, MDD, or PTSD. Results of all multilevel logistic regressions can be found in Table 2 for the probability of a diagnosis at post-treatment and at follow-up. Across all studies, the average probability of a diagnosis was 0.14 (95% CI: [0.05; 0.31]). This indicates that the likelihood of a diagnosis markedly reduced after treatment (before treatment all cases were diagnosed with an internalizing disorder). However, the between-study variance was rather large (Table 2) resulting in a 95% prediction interval for the study-specific probabilities of a diagnosis between 0.0006 and 0.97. This shows that studies varied to a large extent in how likely cases had a diagnosis at post-treatment. At follow-up, the average probability of a diagnosis was 0.12 (95% CI: [0.04; 0.28]). The between-study variance was also large for follow-up (Table 2) with the 95% prediction interval for the random study effects ranging between 0.001 and 0.93.

**Table 2:** Outcome of the two-level logistic regressions estimating the probability of diagnosis

Model	Probability of a diagnosis post-treatment [CI]		Between study variance	ICC	Probability of a diagnosis follow-up [CI]	Between study variance	ICC
Intercept model	Overall	0.14 [0.05–0.31]	7.04	0.68	0.12 [0.04–0.28]	4.12	0.56
Age as predictor	Average age	0.12 [0.04–0.32]	7.91	0.71	0.12 [0.03–0.34]	5.44	0.62
	Average age + one SD	0.12 [0.10–0.14]			0.14 [0.11–0.18]		
Target group as predictor	child	0.12 [0.03–0.37]	7.10	0.68	0.21 [0.06–0.52]	4.19	0.56
	parent	0.03 [0.002–0.40]			0.16 [0.01–0.73]		
	child + parent	0.18 [0.06–0.43]			0.07 [0.02–0.25]		
Disorder category as predictor	AD	0.21 [0.09–0.43]	6.73	0.67	0.14 [0.05–0.32]	3.77	0.53
	MDD	0.02 [0.001–0.22]			0.08 [0.007–0.51]		
	PTSD	0.05 [0.005–0.38]			0.09 [0.01–0.58]		

Variance values are on a logit scale. CI = 95% confidence interval

Age does not seem to have an effect on the diagnosis probability, both for post-treatment and follow-up (*p* = 0.65 and *p* = 0.17; also see Tables 2). The same holds for the variables target group (*p* = 0.48 and *p* = 0.38, respectively) and disorder category (*p* = 0.08 for post-treatment and *p* = 0.85 for follow-up). Still, the average likelihood of a diagnosis at post-treatment and follow-up is indicated to be below chance also when age, target group, or disorder category is taken into account, see Table 2. The large between-study variances and the large intra-class correlations (Table 2) for both post-treatment and follow-up models indicate that a considerable part of the variation in the diagnosis probability is due to differences between studies.

## Discussion

This study is, to our knowledge, the first study to investigate meta-analytically within-person changes instead of between-person differences for evaluating the treatment of youth mental health problems. Evidence for symptom reduction during the treatment phase in comparison to baseline phase was found. Although already during the baseline a slight decrease in symptom severity over time was observed, a larger decrease was found during the treatment phase across cases and studies (Fig. 2). Further, we showed that these within-person treatment effects were positive for the majority of cases but still varied to a large extent between studies and cases. Regarding potential moderators, the immediate decrease in symptom severity of cases with PTSD at treatment start seemed to be more evident compared to cases with AD or MDD. Overall, large improvements at post-treatment and even larger at follow-up were observed for the change in diagnostic status. After the treatments, the likelihood of having an internalizing diagnosis markedly decreased as opposed to the start of the treatment.

Our results indicate that, overall, treatments for internalizing disorders in youths as evaluated in quantitative single-case research seem to be effective in reducing clinical symptoms during treatment. In addition, a positive change in the diagnostic status was observed. These findings are a valuable addition to previous knowledge on treatment effects for internalizing problems in youth from (meta-analyses) of RCTs as they are based on within-person comparisons and tested in a wide range of individuals. These positive results are informative from the clinical point of view as the majority of the sample concerned referred cases that are in general characterized by high severity and comorbidity and harder to be treated. This hypothesis was not quantitatively tested in this meta-analysis, but our impression of the treatments utilized in included studies is that at least in the half of the studies treatments were tailored to some client characteristic. For example, in some studies, treatments were tailored to a specific age group (e.g., young children, Choate et al., 2005; adolescents, Leigh & Clark, 2016), condition (e.g., comorbid AD and ADHD; Jarrett & Ollendick, 2012), or symptom (e.g., behavioral treatment of MDD, Frame et al., 1982). Potentially, this may have impacted the positive results. In addition, this might have also perhaps influenced rather low dropout rates of cases in the studies included in our meta-analysis.

One of the most important findings concerns the ‘variability’ of case characteristics and individual treatment effects. Our results showed that cases and studies are very heterogeneous; there are differences between the cases (within the same study) in demographics, and differences between the studies in designs, type of treatments, number of sessions, and length of treatment (Supp3). In line with this, although overall positive, treatment effects largely varied between cases and studies (Figs. 2 and 3). This emphasizes that variability is a legitime concern in youth intervention research. Worringly, this individual variability has potentially been overlooked in group-level studies. By meta-analyzing single-case studies, we could, for the first time, quantify and describe this heterogeneity in individual treatment effects. In our study, youths with PTSD experienced the most immediate improvement in symptom reduction at treatment start as opposed to baseline when compared to youth with AD or MDD. No other moderating effects were found. This is probably due to various other, not investigated variables that introduced heterogeneity between studies, cases, and treatment effects. Further, the number of studies and, thus, the statistical power, were potentially too low to assess more fine-grained moderator effects.

Next to the limited amount of studies, a notable limitation of our study is that, in graph data analysis, both child and other (parent, observer) report of the outcome variable were included, based on the availability in the studies. It seems that more behavioral symptoms were always rated by others in the included studies. Despite different reporters, the current meta-analysis offers the first overview of quantitative single-case research on internalizing youth and provides empirical evidence for an overall positive within-person treatment effect and considerate heterogeneity between studies and cases for this treatment effects. With the surge of single-case research in internalizing youth, future meta-analyzes will be able to better evaluate moderators explaining the variability of individual treatment effects.

It is worthwhile mentioning that the overall quality of included studies was rated as below average, although there were differences between the studies in quality scores (Supp3). Our general impression was that at least in some studies criteria *were* fulfilled (such as quality criterium ‘treatment adherence’), but this information was not explicitly reported in the specific article. While high-quality guidelines exist for conducting (What Works Clearinghouse, [Kratochwill et al., 2010]) and reporting (Tate et al., 2016) single-case research, much of these guidelines seem left unused in single-case research. The major problems that hinder the utilization seem to be different interpretations of the criteria and the absence of clear procedures for the application of these standards (Maggin et al., 2013). In addition, specific guidelines are necessary for conducting single-case studies in different contexts (laboratory research vs. real-world research), and the designs should be tailored to research questions and aims of the studies, also to increase uniformity of different studies and their generalizability.

In sum, this is, as far as we know, the first study that explored the generalizability of treatment effects found in single-case research in youth treatment outcome studies by meta-analyzing during-treatment within-person changes. Overall, a positive impact of treatments was found for youth-internalizing disorders and symptoms, and the estimated effect was positive for the majority of cases and studies. Yet we also found a large variability between studies and cases in their characteristics and treatment effects. While it is yet to be determined what exactly explains the variation in effects, it is certain that the treatments as evaluated in single-case research hold great clinical potential for youth with mental health problems, and that recent advances in idiosyncratic research methods can help optimally learn what helps for whom and through which mechanisms.

## Appendix 1: Studies Included in the Meta-analysis

- Barterian, J. A., Sanchez, J. M., Magen, J., Siroky, A. K., Mash, B. L., & Carlson, J. S. (2018). An examination of fluoxetine for the treatment of selective mutism using a nonconcurrent multiple-baseline single-case design across 5 cases. *Journal of Psychiatric Practice®*, *24*(1), 2-14.
- Bechor, M., Pettit, J. W., Silverman, W. K., Bar-Haim, Y., Abend, R., Pine, D. S., ... & Jaccard, J. (2014). Attention bias modification treatment for children with anxiety disorders who do not respond to cognitive behavioral therapy: A case series. *Journal of Anxiety Disorders, 28*(2), 154-159.
- Bowyer, L., Wallis, J., & Lee, D. (2014). Developing a compassionate mind to enhance trauma-focused CBT with an adolescent female: A case study. *Behavioural and Cognitive Psychotherapy, 42*(2), 248-254.
- Carlson, J. S., Kratochwill, T. R., & Johnston, H. F. (1999). Sertraline treatment of 5 children diagnosed with selective mutism: A single-case research trial. *Journal of Child and Adolescent Psychopharmacology, 9*(4), 293-306.
- Chevalier, L. L. (2020). Evaluation of a treatment of sleep-related problems in children with anxiety using a multiple baseline design (Doctoral dissertation, Boston University).
- Choate, M. L., Pincus, D. B., Eyberg, S. M., & Barlow, D. H. (2005). Parent-child interaction therapy for treatment of separation anxiety disorder in young children: A pilot study. *Cognitive and Behavioral Practice, 12*(1), 126-135.
- Chorpita, B. F., Albano, A. M., Heimberg, R. G., & Barlow, D. H. (1996). A systematic replication of the prescriptive treatment of school refusal behavior in a single subject. Journal of *Behavior Therapy and Experimental Psychiatry, 27*(3), 281-290.
- Cooper-Vince, C. E., Chou, T., Furr, J. M., Puliafico, A. C., & Comer, J. S. (2016). Videoteleconferencing early child anxiety treatment: A case study of the internet-delivered PCIT CALM (I-CALM) program. *Evidence-Based Practice in Child and Adolescent Mental Health, 1*(1), 24-39.
- Cowart, M. J., & Ollendick, T. H. (2011). Attention training in socially anxious children: a multiple baseline design analysis. *Journal of Anxiety Disorders, 25*(7), 972-977.
- Cunningham, M. J., Wuthrich, V. M., Rapee, R. M., Lyneham, H. J., Schniering, C. A., & Hudson, J. L. (2009). The Cool Teens CD-ROM for anxiety disorders in adolescents. *European Child & Adolescent Psychiatry, 18*(2), 125-129.
- Eckshtain, D., & Gaynor, S. T. (2009). Assessing outcome in cognitive behavior therapy for child depression: An illustrative case series. *Child & Family Behavior Therapy, 31*(2), 94-116.
- Ehrenreich, J. T., Goldstein, C. R., Wright, L. R., & Barlow, D. H. (2009). Development of a unified protocol for the treatment of emotional disorders in youth. *Child & Family Behavior Therapy, 31*(1), 20-37.
- Ehrenreich-May, J., Simpson, G., Stewart, L. M., Kennedy, S. M., Rowley, A. N., Beaumont, A., ... & Wood, J. J. (2020). Treatment of anxiety in older adolescents and young adults with autism spectrum disorders: A pilot study. *Bulletin of the Menninger Clinic, 84*(2), 105-136.
- Eisen, A. R., & Silverman, W. K. (1998). Prescriptive treatment for generalized anxiety disorder in children. *Behavior Therapy, 29*(1), 105-121.
- Eisen, A. R., Raleigh, H., & Neuhoff, C. C. (2008). The unique impact of parent training for separation anxiety disorder in children. *Behavior Therapy, 39*(2), 195-206.
- Esveldt-Dawson, K., Wisner, K. L., Unis, A. S., Matson, J. L., & Kazdin, A. E. (1982). Treatment of phobias in a hospitalized child. *Journal of Behavior Therapy and Experimental Psychiatry, 13*(1), 77-83.
- Farrell, S. P., Hains, A. A., & Davies, W. H. (1998). Cognitive behavioral interventions for sexually abused children exhibiting PTSD symptomatology. *Behavior Therapy, 29*(2), 241-255.
- Farrell, L. J., Kershaw, H., & Ollendick, T. (2018). Play-modified one-session treatment for young children with a specific phobia of dogs: a multiple baseline case series. *Child Psychiatry & Human Development, 49*(2), 317-329.
- Farrell, L. J., Miyamoto, T., Donovan, C. L., Waters, A. M., Krisch, K. A., & Ollendick, T. H. (2021). Virtual reality one-session treatment of child-specific phobia of dogs: A controlled, multiple baseline case series. *Behavior Therapy, 52*(2), 478-491.
- Feather, J. S. (2006). Trauma-focused cognitive behavioural therapy for abused children with. *New Zealand Journal of Psychology, 35*(3).
- Feather, J. S., & Ronan, K. R. (2009). Trauma‐focused CBT with maltreated children: A clinic‐based evaluation of a new treatment manual. *Australian Psychologist, 44*(3), 174-194.
- Fernandez, S., DeMarni Cromer, L., Borntrager, C., Swopes, R., Hanson, R. F., & Davis, J. L. (2013). A case series: Cognitive-behavioral treatment (exposure, relaxation, and rescripting therapy) of trauma-related nightmares experienced by children. *Clinical Case Studies, 12*(1), 39-59.
- Frame, C., Matson, J. L., Sonis, W. A., Fialkov, M. J., & Kazdin, A. E. (1982). Behavioral treatment of depression in a prepubertal child. *Journal of Behavior Therapy and Experimental Psychiatry, 13*(3), 239-243.
- Francis, D., Hudson, J. L., Kohnen, S., Mobach, L., & McArthur, G. M. (2021). The effect of an integrated reading and anxiety intervention for poor readers with anxiety. *PeerJ, 9*, e10987.
- Gaynor, S. T., & Harris, A. (2008). Single-participant assessment of treatment mediators: Strategy description and examples from a behavioral activation intervention for depressed adolescents. *Behavior Modification, 32*(3), 372-402.
- Geuke, G. G., Maric, M., Miočević, M., Wolters, L. H., & de Haan, E. (2019). Testing mediators of youth intervention outcomes using single‐case experimental designs. *New directions for child and adolescent development,* 2019(167), 39-64.
- Girling-Butcher, R. D., & Ronan, K. R. (2009). Brief cognitive-behavioural therapy for children with anxiety disorders: Initial evaluation of a program designed for clinic settings. *Behaviour Change, 26*(1), 27-53.
- Goodall, B., Chadwick, I., McKinnon, A., Werner‐Seidler, A., Meiser‐Stedman, R., Smith, P., & Dalgleish, T. (2017). Translating the cognitive model of PTSD to the treatment of very young children: A single case study of an 8‐year‐old motor vehicle accident survivor. *Journal of Clinical Psychology, 73*(5), 511-523.
- Hagopian, L. P., & Slifer, K. J. (1993). Treatment of separation anxiety disorder with graduated exposure and reinforcement targeting school attendance: A controlled case study. *Journal of Anxiety Disorders, 7*(3), 271-280.
- Heard, P. M., Dadds, M. R., & Conrad, P. (1992). Assessment and treatment of simple phobias in children: Effects on family and marital relationships. *Behaviour Change*, *9*(2), 73-82.
- Hendriks, L., de Kleine, R. A., Heyvaert, M., Becker, E. S., Hendriks, G. J., & van Minnen, A. (2017). Intensive prolonged exposure treatment for adolescent complex posttraumatic stress disorder: a single‐trial design. *Journal of Child Psychology and Psychiatry, 58*(11), 1229-1238.
- Howard, B. L., & Kendall, P. C. (1996). Cognitive-behavioral family therapy for anxiety-disordered children: A multiple-baseline evaluation. *Cognitive Therapy and Research, 20*(5), 423-443.
- Jacob, M., L. Keeley, M., Ritschel, L., & Craighead, W. E. (2013). Behavioural activation for the treatment of low‐income, African American adolescents with major depressive disorder: a case series. *Clinical Psychology & Psychotherapy, 20*(1), 87-96.
- Jarrett, M. A., & Ollendick, T. H. (2012). Treatment of comorbid attention-deficit/hyperactivity disorder and anxiety in children: A multiple baseline design analysis. *Journal of Consulting and Clinical Psychology, 80*(2), 239.
- Kane, M. T., & Kendall, P. C. (1989). Anxiety disorders in children: A multiple-baseline evaluation of a cognitive-behavioral treatment. *Behavior Therapy, 20*(4), 499-508.
- Leger, E., Ladouceur, R., Dugas, M. J., & Freeston, M. H. (2003). Cognitive-behavioral treatment of generalized anxiety disorder among adolescents: A case series. *Journal of the American Academy of Child & Adolescent Psychiatry, 42*(3), 327-330.
- Leigh, E., & Clark, D. M. (2016). Cognitive therapy for social anxiety disorder in adolescents: a development case series. *Behavioural and Cognitive Psychotherapy, 44*(1), 1-17.
- Lewis, K. M., Amatya, K., Coffman, M. F., & Ollendick, T. H. (2015). Treating nighttime fears in young children with bibliotherapy: Evaluating anxiety symptoms and monitoring behavior change. *Journal of Anxiety Disorders, 30*, 103-112.
- Lumpkin, P. W., Silverman, W. K., Weems, C. F., Markham, M. R., & Kurtines, W. M. (2002). Treating a heterogeneous set of anxiety disorders in youths with group cognitive behavioral therapy: A partially nonconcurrent multiple-baseline evaluation. *Behavior Therapy, 33*(1), 163-177.
- Malboeuf-Hurtubise, C., Lacourse, E., Herba, C., Taylor, G., & Amor, L. B. (2017). Mindfulness-based intervention in elementary school students with anxiety and depression: a series of n-of-1 trials on effects and feasibility. *Journal of Evidence-Based Complementary & Alternative Medicine*, *22*(4), 856-869.
- Maric, M., De Haan, E., Hogendoorn, S. M., Wolters, L. H., & Huizenga, H. M. (2015). Evaluating statistical and clinical significance of intervention effects in single-case. experimental designs: An SPSS method to analyze univariate data. *Behavior Therapy*, *46*(2), 230–241.
- Mayer-Brien, S., Turgeon, L., & Lanovaz, M. J. (2017). Effects of a parent training programme for the treatment of young children with separation anxiety disorder. *The Cognitive Behaviour Therapist, 10*.
- Nakamura, B. J., Pestle, S. L., & Chorpita, B. F. (2009). Differential sequencing of cognitive-behavioral techniques for reducing child and adolescent anxiety. *Journal of Cognitive Psychotherapy, 23*(2), 114-135.
- Nelissen, I., Muris, P., & Merckelbach, H. (1995). Computerized exposure and in vivo exposure treatments of spider fear in children: Two case reports. *Journal of Behavior Therapy and Experimental Psychiatry*, *26*(2), 153-156.
- Neuhoff, C. C. (2006). Prescriptive treatment for separation anxiety disorder: child therapy versus parent training (Doctoral dissertation, Fairleigh Dickinson University).
- O'Reilly, M., McNally, D., Sigafoos, J., Lancioni, G. E., Green, V., Edrisinha, C., ... & Didden, R. (2008). Examination of a social problem-solving intervention to treat selective mutism. *Behavior Modification, 32*(2), 182-195.
- Oar, E. L., Farrell, L. J., & Ollendick, T. H. (2015). One session treatment for specific phobias: An adaptation for paediatric blood–injection–injury phobia in youth. *Clinical Child and Family Psychology Review, 18*(4), 370-394.
- Olivier, E., de Roos, C., & Bexkens, A. (2021). Eye movement desensitization and reprocessing in young children (ages 4–8) with posttraumatic stress disorder: A multiple-baseline evaluation. *Child Psychiatry & Human Development*, *53*, 1391-1404.
- Ollendick, T. H. (1995). Cognitive behavioral treatment of panic disorder with agoraphobia in adolescents: A multiple baseline design analysis. *Behavior Therapy*, *26*(3), 517-531.
- Ollendick, T. H., Hagopian, L. P., & Huntzinger, R. M. (1991). Cognitive-behavior therapy with nighttime fearful children. Journal of Behavior Therapy and Experimental Psychiatry, 22(2), 113-121.
- Ollendick, T., Muskett, A., Radtke, S. R., & Smith, I. (2021). Adaptation of one-session treatment for specific phobias for children with autism spectrum disorder using a non-concurrent multiple baseline design: A preliminary investigation. *Journal of Autism and Developmental Disorders, 51*(4), 1015-1027.
- Ortega, M. L. (2012). The generalization of verbal speech across multiple settings for children with selective mutism: A multiple-baseline design pilot study (Doctoral dissertation). Available from ProQuest Dissertations & Theses Global database. (UMI No. 3460734).
- Ooi, Y. P., Raja, M., Sung, S. C., Fung, D. S., & Koh, J. B. (2012). Application of a web-based cognitive-behavioural therapy programme for the treatment of selective mutism in Singapore: a case series study*. Singapore Medical Journal, 53*(7), 446-450.
- Pasquinelli, S. (2009). *The efficacy of treating adolescent depression with Interpersonal Psychotherapy for Adolescents (IPT-A) in the school setting* (Doctoral. Dissertation, Duquesne University, Pittsburg, USA). Retrieved from: https://dsc.duq.edu/etd/1024/.
- Pathak, S., Johns, E. S., & Kowatch, R. A. (2005). Adjunctive quetiapine for treatment-resistant adolescent major depressive disorder: A case series. *Journal of Child & Adolescent Psychopharmacology, 15*(4), 696-702.
- Quero, S., Nebot, S., Rasal, P., Breton-Lopez, J., Banos, R. M., & Botella, C. (2014). Information and communication technologies in the treatment of small animals phobia in childhood. *Behavioral Psychology-Psicología Conductual, 22*(2), 257-276.
- Radtke, S. R., Muskett, A., Coffman, M. F., & Ollendick, T. H. (2022). Bibliotherapy for specific phobias of dogs in young children: a pilot study. *Journal of child and family studies*, *32*, 373-383.
- Ravid, A., Lagbas, E., Johnson, M., & Osborne, T. L. (2021). Targeting co-sleeping in children with anxiety disorders using a modified bedtime pass intervention: A case series using a hanging criterion design. *Behavior Therapy, 52*(2), 298-312.
- Reuland, M. M., & Teachman, B. A. (2014). Interpretation bias modification for youth and their parents: A novel treatment for early adolescent social anxiety. *Journal of Anxiety Disorders*, 28(8), 851-864.
- Ruiz García, A., & Valero Aguayo, L. (2020). INTERVENCIÓN MEDIANTE EXPOSICIÓN MULTIMEDIA EN UN CASO DE FOBIA INFANTIL A LAS AVISPAS. *Behavioral Psychology/Psicología Conductual, 28*(2).
- Saigh, P. A. (1987). In vitro flooding of childhood posttraumatic stress disorders: A systematic replication. Professional School Psychology, 2(2), 135.
- Salazar, D. M., Ruiz, F. J., Ramírez, E. S., & Cardona-Betancourt, V. (2020). Acceptance and commitment therapy focused on repetitive negative thinking for child depression: a randomized multiple-baseline evaluation. *The Psychological Record, 70*(3), 373-386.
- Santucci, L. C., Ehrenreich, J. T., Trosper, S. E., Bennett, S. M., & Pincus, D. B. (2009). Development and preliminary evaluation of a one-week summer treatment program for separation anxiety disorder. *Cognitive and Behavioral Practice, 16*(3), 317-331.
- Simons, M., & Vloet, T. D. (2018). Emetophobia–a metacognitive therapeutic approach for an overlooked disorder. *Zeitschrift für Kinder-und Jugendpsychiatrie und Psychotherapie, 46*, 57-66*.*
- Storch, E. A., Nadeau, J. M., Rudy, B., Collier, A. B., Arnold, E. B., Lewin, A. B., ... & Murphy, T. K. (2015). A case series of cognitive-behavioral therapy augmentation of antidepressant medication for anxiety in children with autism spectrum disorders. *Children's Health Care, 44*(2), 183-198.
- Suveg, C., Kendall, P. C., Comer, J. S., & Robin, J. (2006). Emotion-focused cognitive-behavioral therapy for anxious youth: A multiple-baseline evaluation. *Journal of Contemporary Psychotherapy*, *36*(2), 77-85.
- Taylor, L. K., & Weems, C. F. (2011). Cognitive-behavior therapy for disaster-exposed youth with posttraumatic stress: results from a multiple-baseline examination. *Behavior Therapy, 42*(3), 349-363.
- Tolin, D. F., Whiting, S., Maltby, N., Diefenbach, G. J., Lothstein, M. A., Hardcastle, S., ... & Gray, K. (2009). Intensive (daily) behavior therapy for school refusal: A multiple baseline case series. *Cognitive and Behavioral Practice, 16*(3), 332-344.
- Unterhitzenberger, J., Eberle-Sejari, R., Rassenhofer, M., Sukale, T., Rosner, R., & Goldbeck, L. (2015). Trauma-focused cognitive behavioral therapy with unaccompanied refugee minors: a case series. *BMC psychiatry, 15*(1), 1-9.
- Waters, A. M., Donaldson, J., & Zimmer-Gembeck, M. J. (2008). Cognitive–behavioural therapy combined with an interpersonal skills component in the treatment of generalised anxiety disorder in adolescent females: A case series. *Behaviour Change, 25*(1), 35-43.
- Yorke, J., Nugent, W., Strand, E., Bolen, R., New, J., & Davis, C. (2013). Equine-assisted therapy and its impact on cortisol levels of children and horses: A pilot study and meta-analysis. *Early Child Development and Care, 183*(7), 874-894.

## Supplementary Information

Below is the link to the electronic supplementary material.

Supplementary file1 (DOCX 13 kb)

## Data Availability

All data generated or analyzed during this study as well as the code for analyses are included in this published article, and its supplementary information files.

## Footnotes

1 Results excluding medication treatment: *b*(phase) = − 0.73, 95% CI = [− 1.19; − 0.27]; *b*(time) =  − 0.13, 95% CI = [− 0.24; − 0.02]; *b* (phase*time) =  − 0.30, 95% CI = [− 0.52; − 0.07]. Results without the animal-assissted therapy: *b* (phase) =  − 0.72, 95% CI = [− 1.17; − 0.27]; *b* (time) =  − 0.12, 95% CI = [− 0.23; − 0.02]; *b*(phase*time) =  − 0.30, 95% CI = [− 0.50; − 0.11]. Results excluding non-experimental studies:: *b* (phase) =  − 0.59, 95% CI = [− 0.99; -0.19]; *b* (time) =  − 0.13, 95% CI = [− 0.23; -0.03]; *b*(phase*time) =  − 0.28, 95% CI = [− 0.52; − 0.05].
