# Supplementary material for: A Multilevel Meta-analysis of Single-Case Research on Interventions for Internalizing Disorders in Children and Adolescents
Source: Clin Child Fam Psychol Rev. 2023 Apr 3;26(2):416–29. doi: 10.1007/s10567-023-00432-9 (PMC10123043; doi:10.1007/s10567-023-00432-9)
Supplement: Supplementary file 1 — Supplementary file1 (DOCX 13 kb) [file 10567_2023_432_MOESM1_ESM.docx]

Supplemental materials Supp 1-Supp 6:

<https://osf.io/9qcwa/?view_only=cef4481baee147459be3875d1dd57623>
